# Detangling knots: the intricate roles of G-quadruplexes in herpesvirus replication

**DOI:** 10.1128/jvi.01788-25

**Published:** 2026-06-11

**Authors:** Shea A. Lowery, Sarah A. Gordon, Ananya Paul, Rachel S. Hill, Alex S. Holehouse, Elizabeth B. Draganova

**Affiliations:** 1 Department of Pediatrics, Emory University School of Medicine 1371, Atlanta, Georgia, USA; 2 Department of Biochemistry, Emory University School of Medicine 1371, Atlanta, Georgia, USA; 3 Department of Biochemistry and Molecular Biophysics, Washington University School of Medicine 7548, St. Louis, Missouri, USA; 4 Center for Biomolecular Condensates (CBC), Washington University in St. Louis 7548, St. Louis, Missouri, USA; Indiana University Bloomington, Bloomington, Indiana, USA

**Keywords:** herpesvirus, G-quadruplex, latency, antiviral, small molecule, G4-ligand

## Abstract

The *Herpesviridae* family comprises complex, double-stranded DNA viruses capable of infecting a variety of hosts, including humans. Human herpesviruses, grouped into three subfamilies (alpha, beta, and gamma), establish lifelong, latent infections in most of the global population. Despite widespread prevalence and the ability to cause disease upon reactivation, no cure exists, and current antivirals fail to eliminate latent infections. Therefore, a better understanding of herpesvirus biology is essential for therapeutic development. Recent studies highlight the importance of non-canonical nucleic acid structures, such as G-quadruplexes (G4s), in modulating herpesvirus lytic and latent infection mechanisms. Interestingly, herpesvirus genomes encode an unusually high number of putative G-quadruplex (PG4) forming sequences compared to other viruses. Although G4 biology has been studied for over two decades—initially in human systems—their dynamic nature in cells complicates mechanistic understanding and therapeutic targeting. Herein, we describe the nuances associated with working with G4s at the cellular level, the known and conserved roles of G4s across human herpesviruses, and what is needed to move the field forward. As the technology used to study G4s advances, so will our understanding of how these unique biological structures contribute to herpesvirus replication, potentially paving the way for novel antivirals, and beyond.

## INTRODUCTION

**Fig 1:**
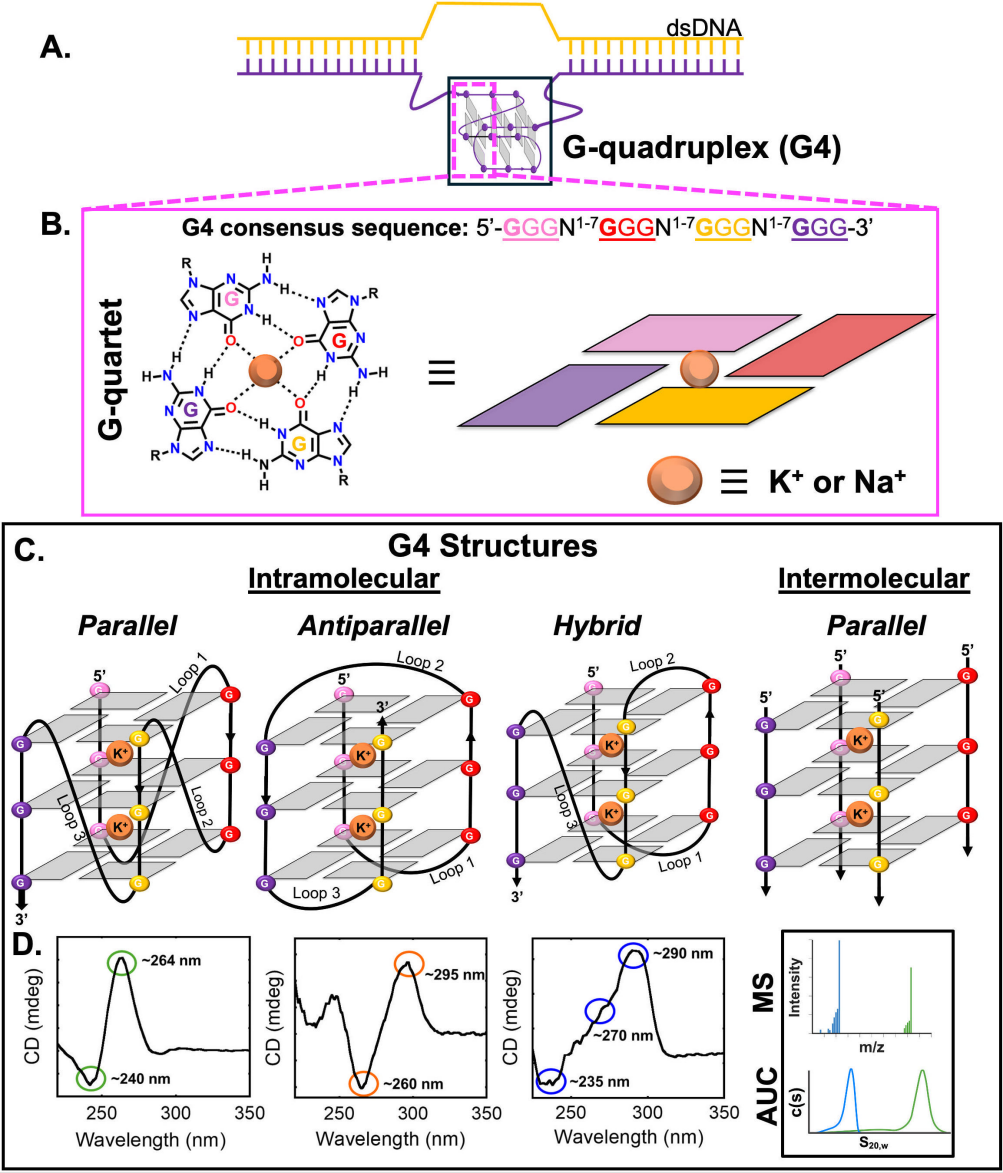
G-quadruplex structure and topology. (**A**) A representative G4 within a dsDNA genome like that of herpesviruses. (**B**) G4 consensus sequence example where the underlined Gs represent each G-tract and bolded Gs participate in one G-quartet; N^1–7^ form the loops shown below. (**C**) Cartoon depiction of intramolecular G4s with either parallel, antiparallel, or hybrid topology and an intermolecular G4. (**D**) Corresponding representative circular dichroism (CD) spectra for each intramolecular topology and either mass spectrometry (MS) or analytical ultracentrifugation (AUC) for an intermolecular G4.

G-quadruplexes (G4s) are non-canonical nucleic acid structures formed through non-Watson-Crick base pairing in G-rich nucleic acid sequences (Fig. 1A and B). G4s were first reported in 1962 (1), followed by connections to human telomere and cancer biology in the early 2000s (2–5). It is well documented that the genomes of eukaryotes, prokaryotes, and viruses contain putative G4 sequences (PG4s) (6–8). Specifically, the human genome is predicted to have thousands of PG4 sequences (9–11) and many of these G4s are involved in fundamental biological processes, including transcriptional regulation, genome stability, telomere biology, and cancer (4, 12–14). Recently, it was shown that all mammalian viruses are predicted to form G4s (7), yet the roles of G4s in viral replication are still largely unknown (15).

Among viruses, the *Herpesviridae* family, the focus of this review, encompasses the most genomic PG4 sequences based on computational predictions (6, 7), which typically analyze G4 consensus sequences (reviewed in reference 16). A standard G4 consensus sequence is represented by G_≥3_N_*x*_G_≥3_N_*x*_G_≥3_N_x_G_≥3_, where multiple G-tracts (series of three or more consecutive guanines; G_≥3_) are separated by varying amounts of other nucleotides (N) (Fig. 1B). G-tract guanines can interact via Hoogsteen hydrogen bonding, forming a G-quartet (Fig. 1B). Three or more quartets can stack onto one another via π-π interactions, producing a DNA or RNA G-quadruplex that is stabilized by monovalent cations such as K^+^ and Na^+^ (Fig. 1A and B) (17).

Herpesviruses are enveloped, ds-DNA viruses that infect a variety of hosts, including mammals, reptiles, and fish (18). The nine known human herpesviruses are grouped into three subfamilies: alpha, beta, and gamma, which vary largely based on cellular tropism (18). Alphaherpesviruses are represented by herpes simplex virus type 1 and 2 (HSV-1 and HSV-2) and varicella zoster virus (VZV) (19, 20). Betaherpesviruses include human cytomegalovirus (HCMV) (21), and human herpesviruses 6A, B, and 7 (HHV-6A/B; HHV-7) (22). Finally, gammaherpesviruses include the oncogenic Epstein-Barr virus (EBV) (23) and Kaposi’s sarcoma-associated herpesvirus (KSHV) (24).

In all instances, herpesviruses establish latent infections, which persist for the lifetime of the host. Currently, herpesviruses infect ~90% of the human population (25). Initial infection and periodic reactivations result in viral spread and various disease states (19). Although particularly detrimental for the immunocompromised, more studies are showing immunocompetent persons are also at risk for severe disease complications with recent connections to Alzheimer’s disease (26), exacerbated COVID-19 symptoms (27), multiple sclerosis (28), and lupus (29). To date, there is no universal cure or vaccine. The only available vaccine prevents VZV (30), and the most commonly prescribed antivirals, acyclovir and derivatives, are most effective against the alphaherpesvirus subfamily, reviewed in reference 31. Recently, maribavir was FDA approved as an HCMV-specific therapeutic (32); however, there remain no antiviral therapies approved for gammaherpesviruses. Therefore, novel prophylactic and therapeutic measures are needed to combat these viruses.

Recent work has demonstrated that all three herpesvirus subfamilies produce G4s with varied functions that contribute to viral replication, and these reports are discussed below. This is not surprising given the large number of PG4 sequences encoded within herpesvirus genomes, yet the molecular mechanisms driving G4 functions in herpesviruses are still largely unknown. Several factors contribute to this knowledge gap. First, the herpesvirus G4 field is relatively new, with initial reports in EBV (2009) (33) and HSV-1 (2015) (34). Second, only a limited number of groups study herpesvirus G4s, with early work focused on alpha- and gammaherpesviruses, yet recent reports of betaherpesvirus G4s are balancing this out (35). Third, technical challenges associated with herpesviruses and G4s in cell culture have led to a predominance of biophysical studies and limited cellular analyses.

Overall, based on the current herpesvirus G4 literature, how G4s operate in both lytic and latent infections—and their potential as antivirals—remains unclear. Herein, we present the currently hypothesized roles of G4s during herpesvirus replication with a focus on the techniques employed to draw these conclusions. We summarize the current bottlenecks in the field of G4 biology and provide suggested best practices moving forward for addressing experimental nuances associated with working with G4s in virally infected cells.

## THE COMPLEXITY OF STUDYING CELLULAR G-QUADRUPLEXES

### G4s are dynamic structures

Recent method advancements shed light on the inherent complexity of G4 structures (36, 37). In cells, G4s are dynamic moieties, folding and unfolding under certain conditions (38, 39). G4s can adopt various topologies, including parallel, antiparallel, and hybrid, formed through either intramolecular (within a single nucleic acid strand) or intermolecular (between two or more strands) interactions (Fig. 1C) (17). It is hypothesized that RNA G4s predominantly adopt a parallel topology due to steric constraints by the 2′-hydroxyl group and evidence from *in vitro* studies, whereas DNA G4 topologies are variable (40). The specific environmental conditions driving G4 topology in a cell are still debated (17, 41). Overall, the dynamic nature of G4s has proven to be a challenging bottleneck for studying G4-related mechanisms or targeting G4s for therapeutic purposes (reviewed in references 12, 13, 42).

### G4 formation, stabilization, and detection

To assess the potential for a G4 sequence to form outside of cellular complexities, researchers typically begin analyses using oligonucleotides containing a PG4. Here, buffers containing K^+^ or Na^+^ can readily induce G4 formation (43) and are confirmed using circular dichroism (CD) spectroscopy (Fig. 1D) (44). CD also distinguishes G4 intramolecular topologies, while mass spectrometry (45) or analytical ultracentrifugation (46) can identify intermolecular topologies (Fig. 1D). NMR and X-ray crystallography can provide 3D structural information yet are technically challenging (47, 48). Nevertheless, these experiments do not confirm if PG4s would form under cellular conditions.

**Fig 2:**
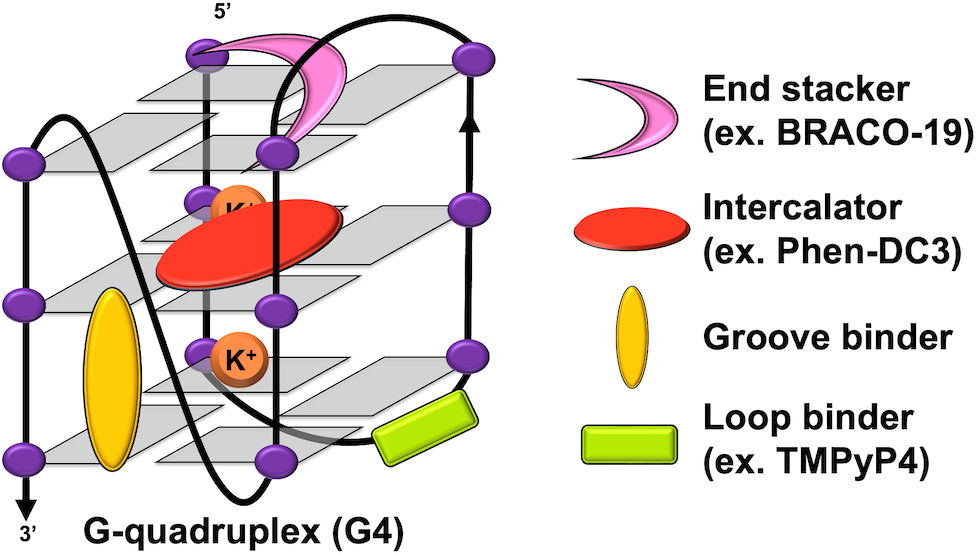
Potential G4-binding interactions with G4 ligands. A schematic model of possible binding sites of G4-interacting ligands. The solution structures of some are reported. PDB IDs: BRACO-19 (PDB: 3CE5), Phen-DC3 (PDB: 7Z9L), and TMPyP4 (PDB: 2HRI). No structural data are available for the groove binder.

To further encourage stable G4 formation, researchers employ G4-stabilizing compounds in the form of small molecules. Here, the stabilizing ligands (Table 1) are added to PG4 oligonucleotides or cells, resulting in the stabilization of G4 structures through various interaction mechanisms (Fig. 2). G4 stabilizers typically share a common chemotype including (i) an aromatic surface that stacks with the external G-quartets, (ii) a side chain with a positive charge or basic groups for G4 groove or loop interactions, and (iii) steric hindrance to prevent intercalation with double-stranded DNA (reviewed in references 49, 50). Common ligands, along with known host and herpes viral target(s) presented in this review, are listed in Table 2 but an exhaustive list of known G4 ligands can be found in the open-access G4 Ligand Database (G4LDB) (51). Nevertheless, G4 ligands do not address if the artificially stabilized G4s will form in cells in the absence of a stabilizer.

**TABLE 1:** Structures and binding modes of commonly used G4 ligands

G4 ligand	Chemotype	Binder mode
BRACO-19 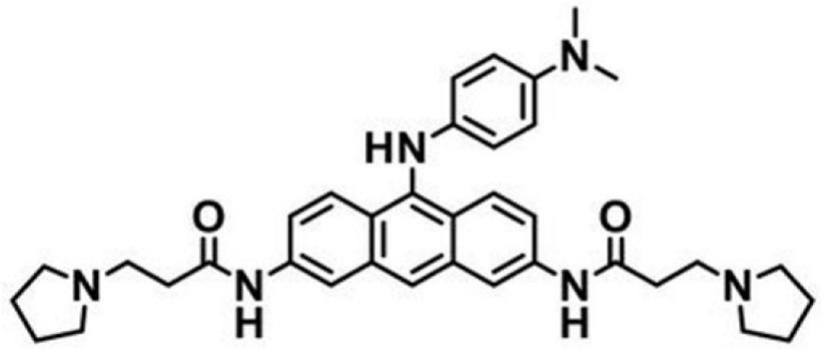	Acridine derivative	End stacking (PDB: 3CE5)
TMPyP2 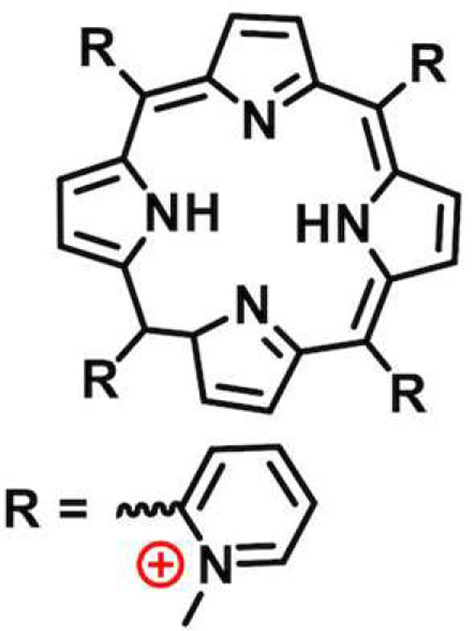	Cationic porphyrin	Expected end stacking and loop binder
TMPyP3 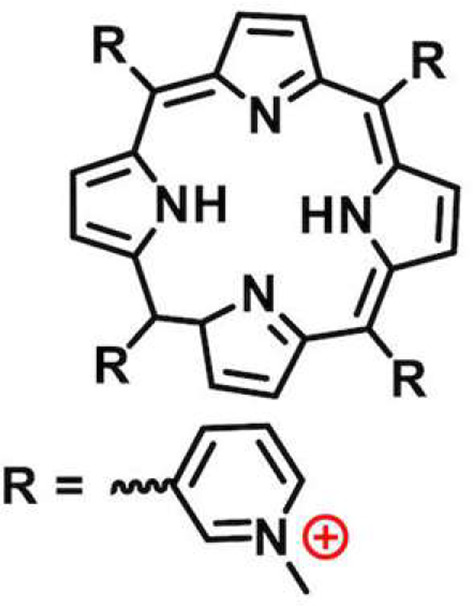	Cationic porphyrin	Expected end stacking and loop binder
TMPyP4 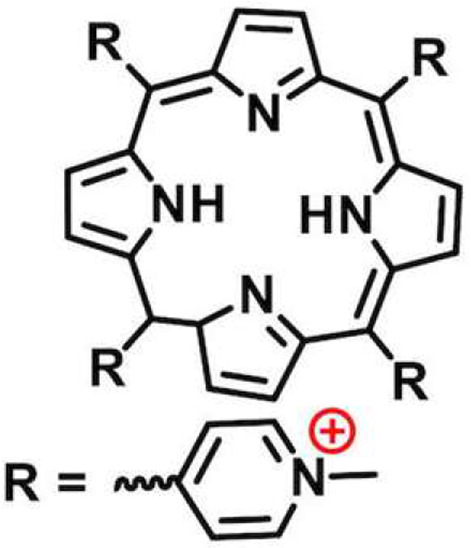	Cationic porphyrin	End stacking and loop binder (PDB: 2HRI)
Phen-DC3 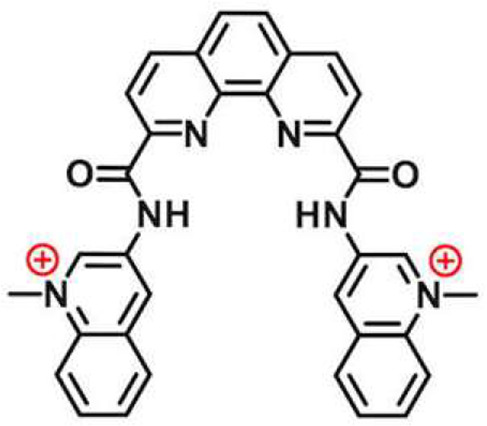	Bisquinolinium- phenanthroline derivative	End stacking (PDB: 2MGN) Intercalator (PDB: 7Z9L)
Phen-DH2 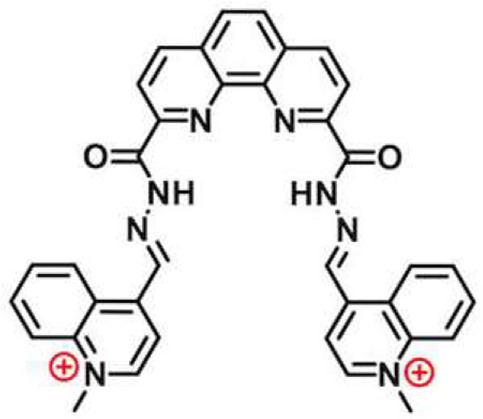	Phenanthroline derivative	Expected to end stack; no structural information
Pyridostatin (PDS) 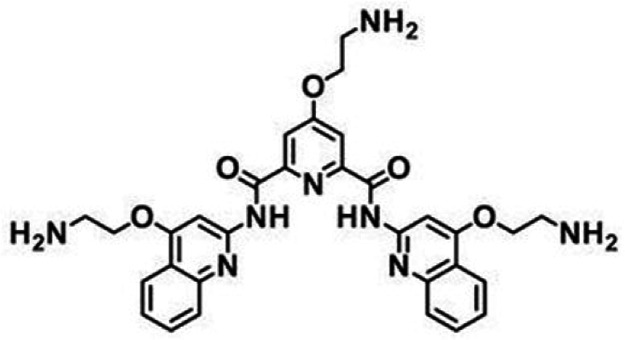	Polyaromatic heterocycle	End stacking (PDB: 7X2Z)
N-Methylmesoporphyrin IX (NMM) 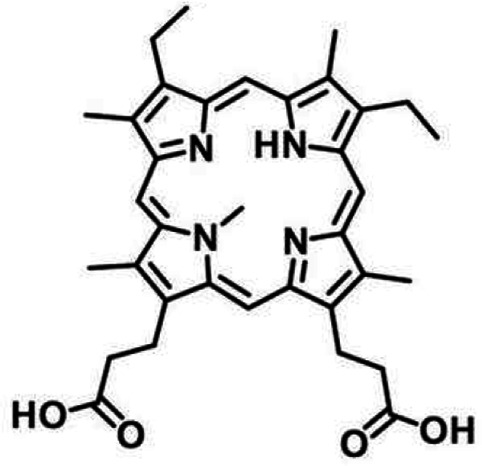	Porphyrin derivative	End stacking (PDB: 4FXM)
GSA-0932 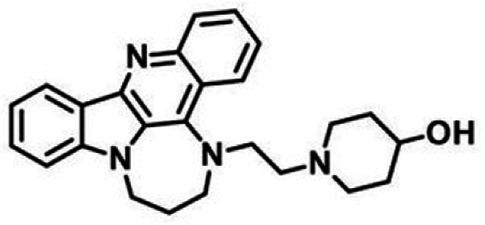	Quindoline derivative	No structural information
c-exNDI2 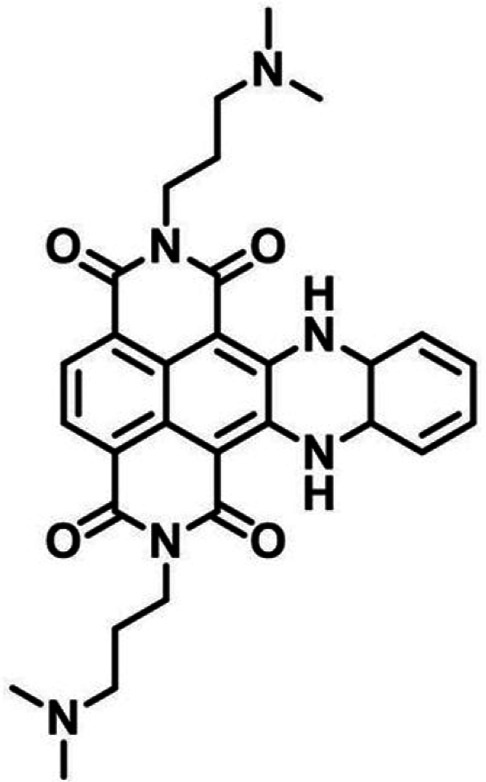	Core-extended naphthalene diimide	Expected to bind as an end stacker
CX-5461 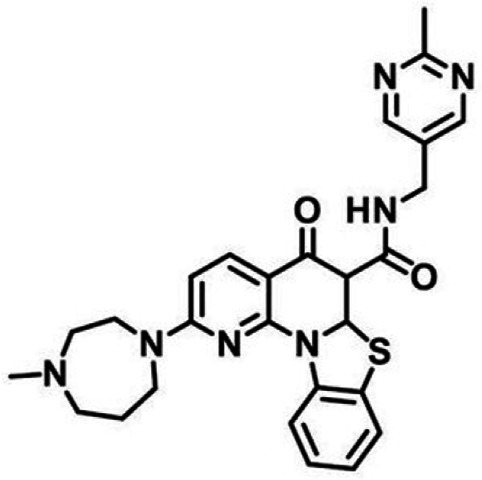	Fluoroquinolone derivative	Expected to bind as end stacking

**TABLE 2:** Commonly used G4 stabilizers in herpesvirus experiments and corresponding phenotypes

G4 ligand	Host G4 target(s)	Virus	Experimental conditions	[Drug] (µM)	Phenotype
BRACO-19	Selectively binds to the human 3′-overhang telomeric G4; inhibits telomerase (52)	HSV-1	*in vitro*	16	Stabilized PG4 oligos (34, 53)
			U2-OS cells transfected with HSV-1 (F) ICP0/27 sequences	5–20	Reduced ICP0 and ICP27 promoter in transfected cells (53)
			U2-OS cells transfected with ICP4 promoter plasmid	5–20	Only ICP4 promoter activity, but not TK, was perturbed (54)
			HSV-1 (F)-infected Vero cells	25	Pretreatment reduced plaque number 35% (34)
				25	2-fold reduction in viral titer when provided between 0 and 8 hpi (time of addition) (34)
				25	Reduced UL36 and UL37 tegument gene expression but not IE (34)
				5–20	Some reduction in vDNA replication (34)
			HSV-1 (F)-infected U2-OS cells	6–50	Reduced ICP4 protein expression at 24 hpi (54)
			HSV-1 (F)-infected HEp-2 cells (HeLa derivative)	25	All five IE genes downregulated at 1.5 hpi; viral genomes not perturbed (55)
		HCMV	*in vitro*	30	No topology changes or increased stability to ER-1 G4 oligo (56)
				IC_50_ = 1.4	Inhibited G4 binding to UL84, but not UL44 or IE2 (56)
			HF cells transfected with HCMV Toledo *ori*Lyt plasmid	5	Reduced OriLyt initiation of DNA replication in transfection assay (56)
		HHV-6	HHV-6A (U1102)-infected U2-OS, HeLa, or MCF-7 cells	1	Reduced HHV-6 integration into host telomeres; no effect on viral entry or vDNA replication (57)
		EBV	EBV-infected Raji Burkitt lymphoma cells	10	Disrupted interaction between EBNA1 and G4 RNA or ORC; reduces EBV genome copies and cell viability (33)
			EBNA1-fusion plasmid transfected into HeLa cells	10	Inhibited EBNA1 metaphase chromosome tethering (33)
TMPyP2	Stabilized human telomeric and promoter DNA G4 (*MYC, KRAS, hTERT*). It also stabilized RNA G4s (58)	HSV-1	*in vitro*	16	Does not stabilize PG4 oligos (34)
			HSV-1 (F)-infected Vero cells	25	Alters virus production at high concentrations; pretreatment reduced plaque number 50%; inhibits viral egress (34)
TMPyP3	Stabilized human telomeric and promoter DNA G4 (*MYC*, *KRAS*, *hTERT*) sequences. Also stabilized RNA G4s (59)	EBV	EBV-infected Raji Burkitt lymphoma cells	10	Disrupted interaction between EBNA1 and G4 RNA or ORC (33)
TMPyP4	Stabilized human telomeric and promoter DNA G4 (*MYC, KRAS, hTERT*) sequences. Also stabilized RNA G4s (52)	HSV-1	HSV-1 (F)-infected Vero cells	0.4	50% plaque reduction (60)
				1-25	Inhibits viral egress, “bags” of virus in cytoplasm (60)
		HCMV	*in vitro*	30	Does not stabilize PG4 oligos strongly (35)
			HF cells transfected with HCMV Toledo *ori*Lyt plasmid	5	Reduced OriLyt initiation of DNA replication in transfection assay (56)
				IC_50_ 0.03–4.31	Inhibits G4 binding by HCMV protein UL84, UL44, and IE2 (106)
		EBV	EBV-infected Raji Burkitt lymphoma cells	10	Disrupts interaction between EBNA1 and G4 RNA or ORC (33)
		KSHV	*in vitro*	10	Stabilized PG4 oligos (33)
			KSHV-infected BCBL-1 cells	10	Altered KSHV episomal DNA replication (61)
			KSHV-infected BCBL-1 cells and KSHV-infected BC3 cells	10	Reduced LANA protein expression by disrupting interaction with hnRNP A1 (62)
			KSHV-positive BCBL-1 cells and KSHV-positive BC3 cells	10	Reduced LANA antigen presentation (62)
Phen-DC3	Stabilized human telomeric sequence and prevented telomerase from binding or extending telomere and telomere uncapping (63)	EBV	EBV-infected B cells (Mutu-1) and EBV-infected cells from nasopharyngeal carcinoma (NPC-6661)	1	Increased EBNA1 expression (64)
			*in vitro*	10	Disrupted interaction between NCL and EBNA1 (64)
		KSHV	*in vitro*	10	Stabilized PG4 oligos (61, 65)
			KSHV-infected BCBL-1 cells	20	Reduced genome copies (61)
Phen-DH2	Unknown interactions in human cells; synthesized for studies on EBV (66)	EBV	EBV-carrying B-cell line 95.8	2	Increased EBNA1 expression (65)
		KSHV	*in vitro*	2	Stabilized oligos corresponding to LANA PG4 oligonucleotides (65)
			FLAG-tagged LANA1 in human H1299 carcinoma-derived cells	2	Increased LANA protein expression (65)
					Induced cytoplasmic relocalization of LANA1 mRNA and disrupts NCL interaction (65)
			KSHV-carrying lymphoma B-cells (BCP-1)	2	Increased LANA expression and LANA antigen presentation (65)
Pyridostatin (PDS)	Targets both DNA telomeric and promoter (c-Myc, KRAS) and RNA G4s. It shows *in vitro* anti-cancer activity (67)	VZV	*in vitro*	30	Slightly stabilizes ORF14/gC PG4 but no topology change (68)
			293T cells transfected with ORF14 plasmid	2.5	Reduced ORF14 mRNA levels (68)
		EBV	*in vitro*	5	Stabilizes PG4 oligos (69)
			293T cells transfected with BHRF1 plasmid	5	Increases BHRF1 mRNA expression (69)
		KSHV	*in vitro*	5	Stabilizes PG4 oligos (69)
			293T cells transfected with v-Bcl-2 plasmid	5	Increased v-Bcl-2 mRNA expression (69)
		HCMV	*in vitro*	5	Did not stabilize PG4 oligos but mildly increased melting temperature (56)
			HF cells transfected with HCMV Toledo *ori*Lyt plasmid	5	Reduced OriLyt initiation of DNA replication in transfection assay (56)
N-Methylmesoporphyrin IX (NMM)	It binds human telomeric G4, DNA promoter gene *SLC2A1*, and detects G4 formed during amyloid-beta (Aβ) fibrillation (70)	KSHV	KSHV-infected BCBL-1 cells	2	Prevents unwinding of G4s by RecQ helicase (71)
			KSHV-infected BCBL-1 cells	2	Reduces viral DNA replication (71)
			KSHV-infected BCBL-1 cells	2	Reduces late gene expression and virion production (71)
			*in vitro*	30	Stabilizes putative G4 oligos (71)
		HCMV	*in vitro*	5	Reduces promoter expression of early and late genes (35)
			HCMV (Toledo) infected HF cells	2.4	Reduces (four log drop) in viral titer (35)
			HCMV (Toledo) infected HF cells	5	Live virus reduced transcripts of UL37, UL35, UL76, and UL146 (IE2) (35, 56, 72)
GSA-0932	Recruits nucleolin (NCL) to the androgen receptor (AR) G4, which suppresses AR gene transcription (73)	HSV-1	*in vitro*	16	Stabilizes putative G4 oligos (74)
			HSV-1 (F)-infected U2-OS cells	EC_50_ = 0.05	GSA-0932 was most potent, 50 nM EC_50_ viral titer (74)
			HSV-1 (F)-infected U2-OS cells	2.5	50% reduction in ICP4 transcript and protein levels (74)
				16	Moderate stabilization of putative G4 oligos (74)
c-exNDI 2	Binds and stabilizes G-rich sequence of MDM2 promoter and shows anti-cancer properties (75)	HSV-1	HSV-1 (F)-infected Vero cells	0.012 and 0.025	Reduced viral titer 50% between 12.5 and 25 nM when provided prior to 12 hpi (76)
CX-5461	Targets telomeric and promoter G4s, *c-KIT1*, and *c-MYC*; Shows anticancer properties by targeting BRCA-deficient cancers (77)	HCMV	HCMV (TB40/E)-infected-MRC-5 cells	0.05	1–2 log reduction in viral titer and viral DNA (78)

The recent development of the G4-specific antibodies 1H6 (79) and BG4 (80, 81), and the nanobodies SG4 (82) and Nb55 (83), confirmed the formation of G4 structures in fixed cells via microscopy (38, 80, 82). In particular, BG4 showed that DNA G4s accumulate within human cell nuclei, peaking during the S phase of the cell cycle and at telomeres (80). In contrast, RNA G4s predominantly form in the cytoplasm, increasing in number during cellular stress conditions (13, 84, 85) and localizing to stress granules (84). Only within the past five years were G4s visualized *in situ,* without fixation, using fluorescently tagged ligands, including PDS (SiR-PyPDS) (38, 86) and the SG4 nanobody (82). Therefore, the study of G4 biology in the context of live cells is only just beginning.

### Mapping G4 sites in oligonucleotides and cells

Over time, groups have developed computational prediction tools, summarized in Table 3, to better identify PG4s based on G4 consensus sequences (reviewed in reference 16), including the commonly used Quadparser (11) and G4Hunter (9, 87). Originally, polymerase stalling assays were used to confirm G4 formation in PG4-oligonucleotides (88). Advances in sequencing technology allowed researchers to employ Illumina Next Generation Sequencing to identify G4 sequences on purified cellular DNA (G4-Seq (89). G4-Seq identified over 700,000 PG4s within the human genome, in line with some computational analyses (9), yet new developments in cellular G4 sequence mapping (discussed below) have since determined human PG4 sequences to be much lower, within the tens of thousands range (90). Furthermore, it is known that G4s form under specific conditions and times, suggesting G4s require a precise cascade to perform an intended function (90). These biological caveats are not taken into consideration in computational prediction tools, although the recent development of G4STAB, a machine learning program for predicting G4 stability based on experimental data (91), suggests this could be developed in the future.

**TABLE 3:** Commonly used methods and considerations for studying G4s in various experimental settings

Experimental system	Tool	Applications	Interpretations	Considerations
**G4 sequence mapping**				
*in silico*	Quadparser (11)	G4-formation prediction based on sequence and sliding windows	Each software predicts propensity for a G4 to form, but under software-specific conditions. Software should be selected based on the biological question to be answered.	Code freely available; can include loops
	G4Hunter (9, 87)	Predicts regions of nucleic acid sequences likely to form G4s. Produces a score based on G-richness and G-skewness; sliding window; commonly used		*Window* (length of sequence to be analyzed): 10-100 bp; 25 bp most common *Threshold* Cutoff value to indicate G4 formation likelihood; 1.4 most common
				Newer version has a web/GUI interface
	QGRS (92)	Similar to Quadparser and G4Hunter as a sliding window approach		Web interface available; can include loops; can directly pull NCBI sequences; can analyze data near RNA processing sites
	G4P Calculator (93)	Sliding window approach		Available for download online
	Quadbase (94)	Searches for G4 patterns		Not as extensive a predictor as other tools; unknown if still freely available
	ImGQfinder (95)	Identifies G4s or *i*-motifs and considers G vacancies, bulges, or mismatches		Code available freely online
	QUFIND (96)	Identifies G4s and CpG islands		Web-based server online
*in vitro*	Polymerase stall assay (34)	Used to identify G4 formation in PG4 oligonucleotides or other nucleic acid sequences	PG4 oligonucleotide sequences are incubated with polymerase for amplification; Loss of polymerase readthrough indicates stalling interpreted as a G4 site	Requires a ligand and/or cation to stabilize a G4, which may not be biologically relevant
	Electrophoretic mobility shift assay (EMSA) (97)	Used to identify proteins with G4-binding capacity using G4 oligonucleotides	Slowing or a shift in gel band(s) indicates oligo is bound to G4 binding protein (e.g., BG4)	Requires cations to stabilize the G4, which may not be biologically relevant
Cellular	ChIP-Seq (90)	G4 sequence mapping in chromatin, particularly in transcriptionally active regions, telomeres, and different promoter sites	Sequence reads from chromatin immunoprecipitation sequencing are indicative of G4 sites	Chromatin preparation, including cross-linking, extraction, and sonication, might affect the endogenous folding of G4 structures
				Assay is in fixed cells
				Antibody bias is a possibility for certain G4 topologies
	CUT&Tag (98)	Enables high-resolution, genome-wide mapping of endogenous DNA using G4 antibody-targeted tagmentation	Sequence reads after tagmentation suggest G4s were formed at these locations	Cell-line dependent reported variations in G4 number are reported, likely due to differences in G4 accessibility and genome sequences
				G4 structures might not be recognized, leading to false negatives, in inaccessible regions
				Untargeted cleavage by Tn5 could result in false positives in highly accessible regions
				G4 antibodies may have non-specific binding artifacts or topology preferences
				Useful method in infected cells to assess if G4 stabilizers prefer viral or host genome sequences
	Chem-map (99)	Uses small-molecule G4 ligands tethered to cleavage or labeling agents to selectively bind G4 and mark nearby DNA for sequencing	Sequence reads after label and cleavage can tell where G4s were formed	Requires small molecules that target G4s
				May give false negative results by missing non-canonical or transient G4s *in vivo*
	G4access (100)	Measures changes in chromatin accessibility at G4-forming regions by combining G4 stabilization with accessibility assays, such as ATAC-like approaches	Provides information on G4 dynamics	Enzyme-related cleaving (MNase) at A/T sequence regions could detect sites that may not always correspond to areas rich in G that can form G4s, potentially leading to false positive results
**Biophysical characterization**				
*in vitro*	Circular dichroism (CD) (44)	Used to characterize G4 topology (e.g., parallel, anti-parallel, and hybrid) of PG4 oligonucleotides	*Parallel* Maximum ≈ 264 nm Minimum ≈ 245 nm *Anti-parallel* Maximum ≈ 295 nm Minimum ≈ 260 nm *Hybrid (3 + 1)*: Maximum ≈ 260 and 295 nm Minimum ≈ 245 nm	Specific buffer recipes are required to encourage G4 formation
				PG4 oligonucleotide G4 sequences may have reduced dynamics compared to genomic sequences
				Cellular conditions, such as molecular crowding, ionic strength, and native helicases, are not present
				G4 stabilizers can readily bind pre-formed G4 oligonucleotides *in vitro*, which may be inaccessible in cells due to differences in environment
	Thermal melting (*T*_m_) assays (101)	Used to assess PG4 oligonucleotide stability in the absence or presence of G4 stabilizers as a proxy for PG4:G4 stabilizer binding	Higher *T*_m_ indicates a more stable G4 Shift in *T*_m_ with ligand interpreted as PG4:G4 stabilizer interactions	Can be monitored via UV-Vis, CD, and Förster resonance energy transfer (FRET)
				Does not always correlate with cellular findings
	Surface plasmon resonance (SPR) (102) and biolayer interferometry (BLI) (103)	Surface-based (BLI) or chip-based (SPR) optical methods for real-time non-covalent interactions of G4 stabilizer interactions with PG4 oligonucleotides	Can measure aspects of PG4 oligonucleotide to G4 stabilizer interactions, including binding affinities, stoichiometry, cooperative effects, in binding, and binding kinetics	Requires specialized instrumentation and consumables
				Can require non-specific binding artifacts to be addressed through buffer screening
**Structural characterization**				
*in vitro*	Nuclear magnetic resonance (NMR) spectroscopy (47)	Used to assess G4 molecular interactions in solution with or without a G4 stabilizer	^1^H NMR spectra: Imino peaks associated with Hoogsteen hydrogen bonds: 10.5–12 ppm 2D NMR methods: ^1^H–^1^H NOESY, ^1^H–^1^H TOCSY, and ^1^H–^1^H COSY ^1^H–^15^N HMBC long-range assignments of G-tetrads	Requires cations to induce G4 formation
	X-ray crystallography (48)	Atomic-level details of G4 interactions, with and without stabilizers	Identifies intermolecular and intramolecular interactions and provides insight into various topologies	Technically intensive technique which requires specialized instrumentation and software
	Mass spectrometry (MS) (104, 105)	Determines G4 formation in oligonucleotides, G4 topological patterns, and number of G-quartets	Changes in *m/z* are used to identify shifts in topology and/or G4 stabilizer binding	Electrospray ionization-MS (ESI-MS) most common
				ESI-MS preserves noncovalent interactions, which typically stabilize the multistranded structures of G4 in solution
	Analytical ultracentrifugation (AUC) (106)	Assesses G4 mass, topology, and G4:G4 stabilizer interactions	Differences in sedimentation can determine G4 mass and topology	Requires specialized equipment
**Transient transfection-based characterization**				
Cellular	Promoter luciferase assay (35, 53)	Determines if a PG4 influences promoter activity upon cloning into a luciferase reporter plasmid	Alteration to luciferase expression levels suggests G4 function at promoter sites is perturbed upon mutation or treatment with G4 stabilizer	Not in the context of an infected cell system
	OriLyt promoter plasmid assay (56)	Determines how G4 stabilizer treatment or mutation of the G4 sequence alters DNA production using OriLyt plasmids	DNA replication is a readout of OriLyt function	Can be adapted for specific herpesvirus OriLyt sites
				Not in the context of an infected cell system
**Virological/antiviral characterization**				
Cellular	Virus mutation (56, 107)	Reverse genetics is used to mutate specific residues in a PG4 sequence to abolish G4 formation in a replicating virus	Reduction in virus production over time suggests mutation of a G4 site reduces replication or abrogates specific interactions	Tedious to generate viruses
				Requires additional validation to ensure phenotype is G4 specific
	Single-step or multi-step growth curves (68, 108)	Assesses virus production over time using either a high (single step) or low (multi-step) MOI in the presence of G4 stabilizer	*Single step*: Altered virus production kinetics at specific lytic replication stages suggest the stage at which G4 stabilizer perturbs *Multi-step*: Reduction in viral spread over multiple rounds of virus replication suggests G4 stabilizer impedes virus replication	Longer time points are required for beta- and gammaherpesviruses
				Not all viruses cause plaques, and instead, a different type of CPE or readout is needed to quantify virus titer
	Time of addition assay (109)	Used to identify the stage of viral replication most affected by a G4 stabilizer when introduced before infection and at various time points post-infection	Time post-infection with the most significant drop in titer suggests G4 stabilizer acts on this aspect of viral replication	Compounds that perturb later stages of infection or produce assembly defects may produce a similar reduction in virus titers at all time points
	Entry assays (110)	Used to measure neutralization, attachment, adsorption, or entry of the virus in the presence of ligand	Identifies if a ligand alters a specific stage of viral entry	Multiple variations in timing of ligand added and changes in temperature change the readout
	qPCR/qRT-PCR (68)	Use of a specific primer allows for quantification of viral genome or a specific gene product	Identifies specific gene product/viral DNA altered upon ligand treatment	Careful time point selection is essential as genes are expressed at different times post-infection
	EC_50_ (99,160)	Determines the G4 stabilizer concentration when viral replication is at half maximum	The EC_50_ is determined after plotting all data points on the curve	Essential antiviral assay for potency
	Cell toxicity assay (111)	Determines cellular toxicity upon G4 stabilizer treatment using a range of concentrations	Reduction in cell metabolism or death is measured with a kit and suggests the compound has some toxicity	Always perform toxicity assays before any other cellular assay
**G4 visualization**				
Cellular	Immunofluorescent (IF) microscopy (56, 80, 112, 113)	Identifies cellular location of G4s using G4 antibodies, such as BG4, 1H6, and SG4	G4 formation resembles fluorescent puncta. Identifies cellular location of G4 and can be quantified per field.	Some concerns for nonspecific binding (BG4) and affinity for different types of G4s. DNase or RNase treatment is often used to remove specific G4s.

### Viruses add complexity to G4 dynamics

Studying viral G4s in cells remains challenging, as both host and viral genomes contain targetable G4 sequences. However, their dynamics may differ, with viral G4s potentially enriched during viral replication, leading to preferential G4 stabilizer targeting. For example, herpesviruses stall host cells in the G_0_/G_1_ phases (114), while host G4s form during the S phase (80), and viral host shutoff can further reduce host G4 formation (115, 116). Nevertheless, as viruses rely on host machinery, modulations to host G4s may also impact viral replication. Overall, the link between *in vitro* G4 stabilization and cellular function remains unclear, and *in vitro* assays may overestimate biological relevance. Emerging tools to study G4 biology described below will help address these complexities.

## ROLES OF G4s IN HERPESVIRUS REPLICATION

### G4s could form at various points during herpesvirus replication

**Fig 3:**
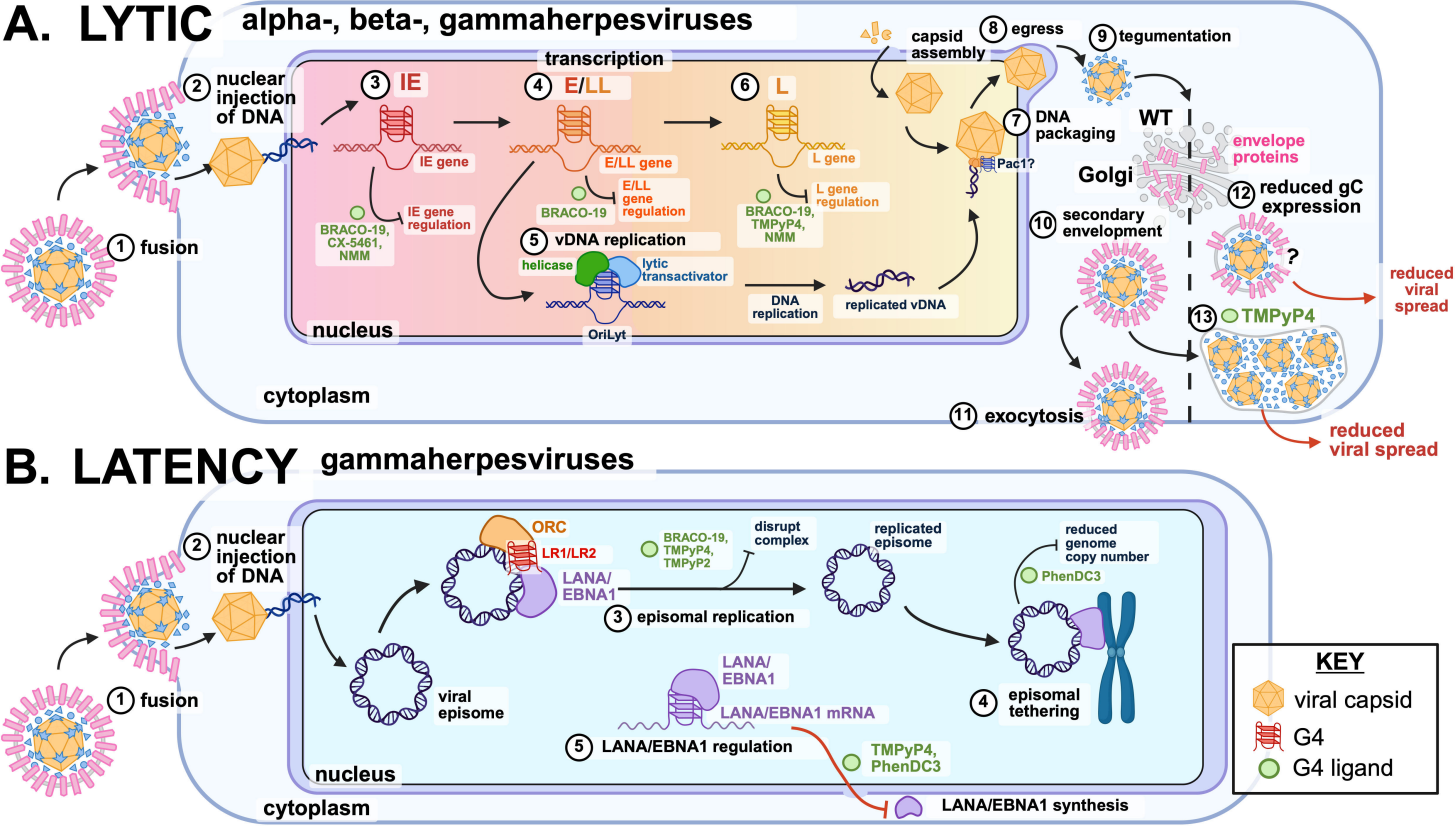
Overview of herpesvirus replication and G4 functions based on G4 ligand studies. (**A**) **Lytic infection:** (**1**) Herpesviruses enter cells through fusion or endocytosis, releasing the capsid for cytoplasmic transport to the nucleus, the site of viral replication; (**2**) upon genome delivery into the nucleus, the virus will enter a lytic or latent state. During lytic infection, viral G4s contribute to (**3**) immediate early (IE) and (**4**) early (E) gene expression. For beta and gammaherpesviruses, direct connections with G4s and (**5**) vDNA replication have also been identified. G4s in (**6**) late (L) gene expression, which follows vDNA replication, are also reported. Next, the genome is (**7**) packaged into assembled capsids, dependent on the genomic Pac1 sequence reported to form a G4 *in vitro*. Capsids undergo (**8**) nuclear egress, followed by (**9**) cytoplasmic tegumentation, and (**10**) secondary envelopment, producing a viral particle which (**11**) exits the cell via exocytosis. Alterations to some viral G4s limit viral spread, either through (**12**) reduced late protein expression or (**13**) unknown mechanisms. (**B**) **Latent infection:** upon establishing latency after virus entry (**1-2**), G4s participate in (**3**) replicating the episome, (**4**) episomal tethering, and (**5**) regulating LANA/EBNA1 expression. In both panels, G4 ligands used to determine the proposed G4 roles are in green. Corresponding experimental details and citations for the effects of the G4 ligands shown in this figure are in Table 2. Figure was made with Biorender.com.

Herpesviruses initially enter cells through fusion or endocytosis, followed by capsid trafficking to the nucleus, the site of genome replication (Fig. 3) (19). In a productive infection (Fig. 3A), genome replication occurs through a controlled cascade in four phases: immediate early (IE), early (E), leaky late (LL), and late (L) (18, 19). Within hours, herpesviruses recruit host and viral machinery to IE promoter regions, resulting in the transcription of the IE genes. IE mRNA is translated in the cytoplasm, kickstarting a cascade of E gene expression, protein production, and viral DNA replication. In turn, LL and L genes are translated, structural proteins are produced, and new virions are formed. In theory, the PG4s seen in non-coding and coding regions of the herpesvirus genome could provide a mechanism for transcriptional control, altering viral gene expression and subsequent steps in the viral replication cycle. Similar implications of G4-mediated gene regulation and genome instability are well documented in cellular systems (reviewed in reference 117) and have been made for herpesviruses (Fig. 3), which we describe below.

### Herpesvirus GC genome content does not correlate with G4 propensity

**Fig 4:**
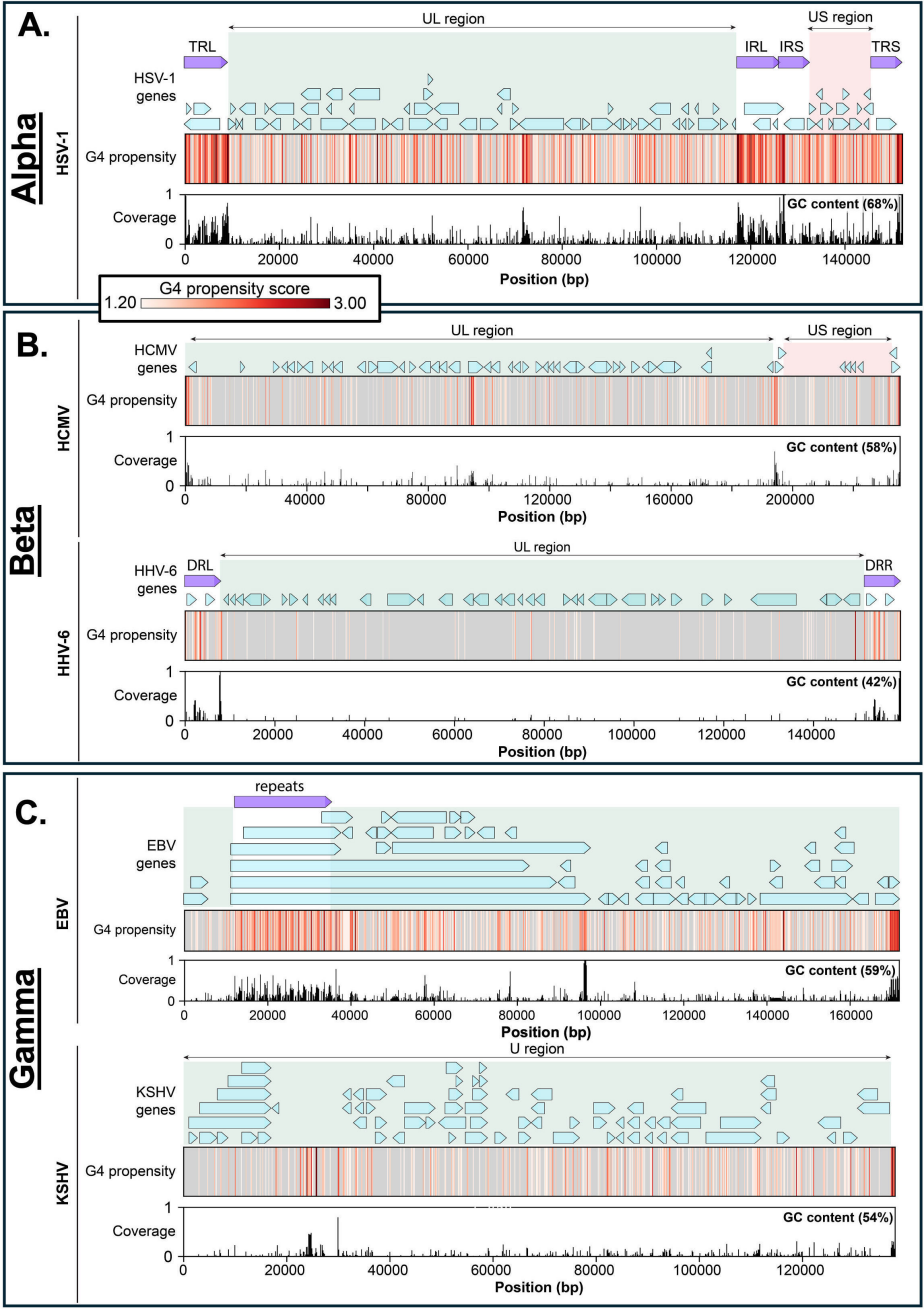
Herpesvirus genomes represented as a heat map for G4-forming propensity. G4-forming propensity was predicted using G4Hunter for the genomes of representative (A) alpha (HSV-1), (B) beta (HCMV; HHV-6A), and (C) gammaherpesviruses (EBV; KSHV) and is displayed as a heatmap. G4Hunter analysis used default parameters for strand-agnostic G4 prediction (25 bp window size; score threshold of 1.2). For heatmaps, the number of bins was set such that each bin across the different sequences consistently holds 152 bps. Corresponding genomic regions (top), along with open reading frames (blue arrows), as well as base pair location within the genome (bottom) are displayed. DRL, direct repeat left; DRR, direct repeat right; IRL/IRS, internal repeat long/short; TR, terminal repeat; TRL/TRS, terminal repeat long/short; UL, unique long; US, unique short. The following sequences were used for analyses: HSV-1 F strain (GenBank: KM222724.1); HCMV Toledo strain (GenBank: GU937742); HHV-6A Variant A (GenBank: NC_001664); EBV B95-8 strain (GenBank: NC_007605); and KSHV GK18 strain (GenBank: NC_009333). The updated G4Hunter code is available on GitHub at https://github.com/holehouse-lab/g4hunterpy3 with detailed documentation at https://g4hunterpy3.readthedocs.io/.

All human herpesvirus genomes are made of linear, double-stranded DNA, ranging from 125 to 250 kbp (118), containing unique regions flanked by inverted repeats (Fig. 4). The total GC content between subfamilies vastly differs with HSV-1 being the highest (68%) (Fig. 4A). It should be noted that GC richness does not correlate with a higher number of PG4 sequences (7, 119). For example, the genomes of HCMV and EBV are similar in GC content levels (58% and 59%, respectively), yet HCMV contains significantly fewer PG4 sequences compared to EBV (Fig. 4B and C). Therefore, herpesvirus genomic G4 sequences are not a result of random events (119) or GC richness, suggesting viral G4s perform specific functions.

### Herpesvirus subfamilies exhibit differences in G4 propensity distribution

Herpesvirus genomes typically contain two coding regions, referred to as unique long (UL) and unique short (US), flanked by non-coding terminal (TR_L_/TR_S_) and inverted repeats (IR_L_/IR_S_) (Fig. 4) (19). Two human herpesvirus exceptions include KSHV and HHV-6, which encode a singular unique long region flanked by repeats (120, 121). The genome sequences of the human herpesviruses were previously assessed by other groups for PG4s using various tools including Quadparser (11, 69, 119), G4Hunter (9, 34, 53, 68, 87, 122), QGRS (53, 61, 92, 123), Quadbase (53, 94), or other software (35). Each software is slightly different (Table 3), and software selection is reviewed elsewhere (16). Here, for consistency, we compiled a heatmap of predicted G4 sequences for representative herpesviruses from genomes among the three subfamilies using G4Hunter updated by our labs for use with Python 3.0 (Fig. 4). Consistent with prior reports (119), HSV-1 is predicted to contain the most PG4 sequences, followed by EBV, KSHV, HCMV, and HHV-6A (Fig. 4).

For all genomes, PG4s predominantly localize to the repeat regions. Furthermore, HSV-1, HCMV, and KSHV PG4s are also enriched at promoter regions for IE genes. The same finding was observed in pseudorabies virus (PRV), an alphaherpesvirus homolog in swine (123). EBV has fewer IE genes than any of the other herpesviruses, so this trend was less clear (119). In HSV-1, PG4s are also found before the consensus motif sequence for VP16 (53), an essential transactivator conserved in alphaherpesviruses. Finally, PG4s are located in the promoter sequences within conserved genes required for DNA replication, capsid assembly, capsid packaging, tegumentation, and egress for all three subfamilies (119).

PG4 patterns within the betaherpesvirus subfamily are the most strikingly different, with a wide distribution of PG4s across the HCMV genome, whereas HHV-6A PG4s localize solely to the repeat regions (Fig. 4B). It is hypothesized HHV-6A PG4s may aid in viral host genome integration, whereas current HCMV PG4 studies focus on initiation of the lytic replication cycle (57, 124) and could explain this discrepancy. One group developed an algorithm to predict conventional, long-loop, and bulge-containing PG4 sequences within HCMV (35). Most were categorized as unconventional, with long or bulged loops between the G-tracts, which form the G4, a similar phenotype observed for VZV (68), and *in vitro*, all tested unconventional sequences formed G4s assessed via CD. It would be interesting to see if other herpesviruses share this feature and if these loop structures have downstream biological purposes.

### Alphaherpesviruses

#### Terminal and inverted repeats

The first characterization of alphaherpesvirus PG4s assessed the topology of oligonucleotides corresponding to the highest-scoring and most repeated PG4 sequences in HSV-1, which mapped to the non-coding IRs and TRs and are in alignment with our analyses (Fig. 4A). Oligonucleotides were designated as “un” if corresponding to a non-coding region, such as TR, or by the corresponding gene name. Here, both the un1 (IR_L_/TR_L_ regions) and un3 (IR/TR_L_/TR_S_ regions) sequences adopted parallel topology, while un2 (IR_S_/TR_S_) adopted antiparallel topology via CD. In contrast, VZV clusters PG4s within the IR_S_ and TR_S_ genomic regions and predominantly adopt hybrid or antiparallel topologies in CD analyses (68). CD and melting temperature assessments also show commercially available stabilizers BRACO-19 (HSV-1), GSA-0932 (HSV-1), and pyridostatin [(PDS); in VZV] (Table 2) enhanced G4 stability *in vitro*, suggesting these sequences could form G4s in cells (34, 68, 74).

To confirm the biological relevance of HSV-1 un2, U2-OS cells, an osteosarcoma cell line permissive to HSV-1 (125, 126), were transfected with a biotinylated DNA probe for the un2 sequence, which does not perturb un2 G4 folding (54). Cells were then infected with HSV-1 and showed fluorescent signals with the 1H6 G4 antibody localized in the nucleus at 8 h post-infection (hpi), suggesting the un2 G4s form during HSV-1 replication. It was further determined that un2 interacts with ICP4 (54), the essential transcriptional regulator for HSV-1 infection (127), discussed below.

#### Immediate early replication processes

In addition to repeat regions, the promoters of all five HSV-1 and HSV-2 IE genes (ICP0, ICP4, ICP22, ICP27, and ICP47) and VZV homologs (ORF61, ORF62, ORF63, and ORF4, respectively) are also rich in PG4s (53, 68). Most oligonucleotides of these PG4s adopt a parallel topology, although some were antiparallel or hybrid. BRACO-19 stabilized the G4 oligonucleotides *in vitro,* suggesting these are G4-forming sequences (53). To support PG4 function at the viral promoters, U2-OS cells were transfected with luciferase expression plasmids encoding promoter sequences for either the ICP0 or ICP27 genes, followed by BRACO-19 treatment. Promoter activity was perturbed in a dose-dependent manner, suggesting G4 stabilization impedes ICP0- and ICP27-mediated transcription *in vitro*. It is unclear if BRACO-19 interacts with all the tested HSV-1 promoter sequences during an active cellular infection or if it exhibits sequence specificity.

HSV-1-infected U2-OS nuclear lysates were incubated with the HSV-1 IR_S_/TR_S_ PG4 un2L2 oligonucleotide (a longer version of the first reported un2 oligonucleotide) and subjected to a pulldown assay followed by mass spectrometry (54). ICP4 was identified as a strong un2L2 binder. Here, the un2L2 oligonucleotide adopted a parallel topology, and it was proposed that *in vitro*, ICP4 preferentially binds and unfolds parallel G4s based on additional *in vitro* CD analyses. This could provide one potential mechanism for how ICP4 could use G4 structures to regulate HSV-1 gene expression, yet to date, there are no reports describing ICP4 as a helicase. Furthermore, although most HSV-1 PG4 sequences characterized *in vitro* adopt a parallel topology, other topologies are also reported (34, 53, 54). It is well documented that environmental conditions can influence G4 topology (128). Therefore, it remains unclear if any of these HSV-1 PG4s exhibit structural polymorphism under altered conditions and if so, how this would impact G4 function.

It was recently shown that cellular ATRX, a component of the promyelocytic leukemia nuclear body, with which HSV-1 genomes associate upon cellular entry, binds IE gene promoters at PG4 sites (55). siRNA knockdown of ATRX, or stabilization of G4s with BRACO-19, resulted in reduced viral gene expression at 1.5 hpi, indicating G4 formation may help ATRX promote IE transcription (55). Further studies are required to understand how ATRX interacts with IE G4s; however, the global downregulation of IE transcripts indicates a broad specificity for IE G4s.

#### Early phase and viral DNA replication processes

Immunofluorescence experiments with the G4-specific antibody 1H6 showed HSV-1 G4s accumulate within the nucleus at 6–14 hpi in Vero cells, moderately colocalizing with the replication compartment marker ICP8 at 6 hpi (112). Only DNase, rather than RNase, reduced fluorescent signals, suggesting the visualized G4s were formed from DNA. This is in line with reports that 1H6 predominantly binds DNA G4s, while BG4 displays cross-reactivity, binding both DNA and RNA G4s (80, 81).

A pulldown assay using HSV-1-infected U2-OS cell nuclear lysates with the HSV-1 PG4 un2L2 (IR_S_/TR_S_) or UL36 (tegument) oligonucleotides identified interactions with the UL42 protein (54)—an essential processivity factor to the viral DNA polymerase (129), but the biological implications of this are unknown. There are limited examples of G4 stabilizers affecting vDNA replication, with only BRACO-19 shown to moderately inhibit HSV-1 vDNA synthesis (34), and more recently by epiberberine, a G4-stabilizer from natural sources (130), which impaired vDNA synthesis in PRV (131). Alternatively, G4s may be involved in viral recombination as HSV-1 PG4 hotspots coincide with known HSV-1 recombination breakpoints (132). Therefore, it is possible G4s are not directly regulating vDNA synthesis in the nucleus but rather aid in the localization of machinery necessary for recombination, yet this awaits further testing.

#### Leaky late and late replication processes

Multiple quadruplex sequences are enriched in conserved viral genes, which produce proteins required for capsid assembly, packaging, and nuclear/cytoplasmic egress (119). G4s have been identified at sites of HSV-1 nuclear capsid egress and cytoplasmic maturation in infected cell TEM (112), yet no studies thus far directly implicate G4s in these processes. The original pulldown assay using HSV-1 G4 oligonucleotide baits against nuclear lysates from HSV-1-infected cells identified the major capsid protein UL19/VP5, and triplexes 1/UL38/VP19c and 2/UL18/VP23 as the main targets for the TR region un2L2 G4 oligo (54). All three proteins are essential for capsid assembly (133), yet why these proteins would bind G4s is unclear.

A PG4 was identified in the Pac1 sequence element (134), which is required for viral genome cleavage and subsequent encapsidation of newly replicated viral DNA into the capsid (135). The Pac1 PG4 forms a G4 *in vitro*, yet it has not been directly implicated in the capsid packaging process (134, 136). Pac1 must be cleaved by the viral terminase complex to insert the viral genome into the capsid. It is possible that G4 stabilization at this genome site could either impact the cleavage of newly replicated viral DNA, serve as a landing site for packaging machinery, or provide steric hindrance to the amount of viral DNA packaged. How these interactions might regulate the timing of genome cleavage and packaging remains to be determined.

Interestingly, treatment of HSV-1-infected Vero cells with the porphyrin-based G4 stabilizer, TMPyP4, reduced supernatant viral titers, and TEM showed HSV-1 particles trapped in vesicles within the cytoplasm (60). Yet DNA replication levels remained normal, suggesting TMPyP4 produces a virion maturation and/or cytoplasmic egress defect. Similarly, the isomer TMPyP2 perturbed cytoplasmic egress, but only at high doses, possibly due to a weaker affinity for G4s compared to TMPyP4 (60, 137). The exact mechanism of action remains to be determined, including the specific G4 sequences TMPyP4 binds to, and presumably stabilizes, that then produce the reported assembly defect.

The VZV ORF14/gC glycoprotein gene sequence contains multiple PG4 sequences, which adopt an anti-parallel topology *in vitro* (68). Cells transfected with a gC-coding plasmid showed reduced gC expression levels when treated with the G4 ligand PDS. Interestingly, gC expression increased when a subset of gC G4 sequences were deleted, resulting in a defect in cellular spread. Overall, these findings are consistent with previously reported G4 mechanisms involved in modulating gene expression in various biological systems (13).

### Betaherpesviruses

#### Promoter-related processes

The HCMV genome overall contains fewer total numbers of PG4 sequences; however, they reside within regulatory, promoter, and coding regions, like that of alphaherpesviruses (Fig. 4A and B). Oligonucleotides corresponding to HCMV promoter sites exhibited characteristic G4 spectral signatures via CD, confirming a potential to form G4s *in vitro* (35, 72). Most sequences adopted a parallel topology, with the exception of a G4 in UL51 (hybrid; HSV-1 UL33 homolog) and UL75 (antiparallel; HSV-1 gH homolog) (35). Two different porphyrin-based G4 stabilizers, N-methyl mesoporphyrin IX (NMM; Tables 1 and 2) and TMPyP4, increased the G4 oligonucleotide melting temperatures without a spectral shift in the CD spectra. A G4 sequence in the miR-US33 promoter was also identified and, as an oligonucleotide, adopted a parallel topology *in vitro*. CD and melting curve analyses showed treatment with TMPyP4 destabilized this oligonucleotide while PDS produced a stabilizing effect (138). Overall, these findings suggest G4-ligand interactions may be not only sequence-specific but also related to topology.

The first HCMV genome-wide promoter PG4 analysis identified 37 PG4s, of which all formed G4s *in vitro*, yet only nine produced a promoter defect when cloned into a luciferase reporter plasmid, transfected into human foreskin fibroblast (HFF) cells, followed by cellular infection with HCMV (35). Treatment with NMM (either at or after infection) altered the activity of the following genes: IE (UL37), E (UL34, UL35, RNA4.9, and UL142), and L (RL6, UL6, UL76, and US29). Activity was not perturbed in the presence of the control ligand, TMPyP2, known to weakly interact with G4s. Further evaluation in HCMV-infected HFF cells showed gene expression was reduced for innate immune modulating genes UL37 (139), UL35 (140), and UL76 (141). Finally, NMM treatment of HCMV-infected cells significantly reduced viral DNA levels, and viral replication was reduced by ~3.5 log indicating NMM is a potent antiviral against HCMV (35).

#### Immediate early and viral DNA replication processes

HCMV G4s have also been investigated in the context of DNA replication. Immunofluorescence experiments with both the 1H6 and BG4 antibodies showed HCMV G4s accumulate in cellular nuclei within replication compartments of HCMV-infected human fibroblast cells (56), similar to HSV-1 (112). Treatment of HCMV-infected MRC-5 fibroblast cells with the CX-5461 G4 ligand, a known RNA Polymerase I inhibitor (142), disrupted viral genome replication and reduced viral titers by ~2 logs (78). The addition of the clinically approved maribavir with CX-5461 enhanced the replication defect in a synergistic manner, suggesting combination therapies should be kept in mind when exploring G4 ligands as antivirals.

Both the essential region-1 (ER-1) and -2 (ER-2) within the HCMV *ori*Lyt site are also predicted to form G4s and oligonucleotides corresponding to those PG4s exhibited a parallel topology aside from one which was antiparallel (56). In cells, only the G4 from the ER-1 site was identified as essential for viral replication, determined using G4-mutant viruses and further corroborated through a series of biochemical analyses. A pulldown assay with the ER-1 G4 DNA identified the replication protein IE2 (transactivator) (143), UL84 (replication initiator and IE2-binding partner) (144), and UL44 (DNA polymerase processivity factor) (145) as potential interacting partners. These proteins displayed higher affinity for the HCMV ER-1 G4 compared to the human cMyc G4, highlighting the specificity of the interaction (56). Furthermore, only specific G4 stabilizers could perturb G4 binding to each of the three proteins, suggesting the respective binding interactions with G4s differ and warrant further investigation.

Most recently, it was found that the IE2 protein transactivates the viral chemokine UL146 promoter, which produces vCXCL1, by interacting with a G4 within the UL146 locus (72). UL146 G4 binding to IE2 and promoter activity could be disrupted by NMM and TMPyP4 but not peimine, a suggested G4 groove binder. Transient transfection assays assessing *ori*Lyt expression also showed G4 ligand specificity (56), again suggesting not all G4 ligands interact in cells the same.

#### Host genome integration

Unlike HCMV, the related betaherpesvirus HHV-6A/B integrates the viral genome into the host, typically at telomeric regions within chromosomal termini (124). A study reported that in the presence of BRACO-19, HHV-6A/B integration events were impaired in either HHV-6A/B-infected HeLa or MCF-7 cells but not U2-OS cells (57). It was proposed that BRACO-19 perturbed telomerase activity, presumably through telomeric G4 stabilization. Telomerase is not produced in U2-OS cells but is present in HeLa and MCF-7 cell lines, explaining how telomerase stalling would prevent elongation and result in premature cellular death in a cell-line specific manner. Interestingly, BRACO-19 did not impede HHV-6A/B viral DNA replication (57); however, these cell lines are not fully permissive to HHV-6 and are cancer-derived; therefore, other permissive primary cell lines should be evaluated in the future regarding viral replication and host genome integration studies. A separate hypothesis is that HHV-6A/B integration events are perturbed in the presence of BRACO-19 due to stabilization of viral PG4s located within TR and IR regions (Fig. 4). This interesting finding awaits further investigation.

### Gammaherpesviruses

The gammaherpesviruses Epstein-Barr virus (EBV) and Kaposi’s sarcoma-associated herpesvirus (KSHV) are unlike the other subfamilies in that both are oncogenic viruses (23, 24). Both viruses establish latency in actively dividing B cells, where the viral genome persists as a circularized episome, ultimately requiring the virus to evade host immune defenses throughout cellular division. The studies below implicate G4s in various aspects of latency maintenance, potentially serving as contributors to overcoming hurdles associated with the virus remaining undetected in actively dividing cells (146). In contrast, the implications of G4s in gammaherpesvirus lytic infection are less defined due to experimental challenges working in cell culture, as gammaherpesviruses immediately enter latency upon cellular infection unless reactivated using chemicals or viral mutagenesis.

### Latent infection

#### Episome persistence

Original reports focused on the EBV nuclear antigen 1 (EBNA-1) protein, which is essential for maintenance and replication of the viral episome during latency (23). EBNA-1 recruits the cellular origin recognition complex (ORC) to the *ori*P site, particularly at G-rich RNA sequences within the EBNA-1 linking regions (LR1 and LR2) (33, 147), each located within larger glycine and arginine-rich domains (148), presumed to be G4s, although this was not tested using CD (33). The G4 stabilizers TMPyP3, TMPyP4, and BRACO-19, but not TMPyP2, prevented ORC association with EBNA-1 *in vitro*, confirming these findings.

In EBV-infected Raji Burkitt lymphoma cells, BRACO-19 reduced the EBV genome copy number and disrupted cellular growth (33). Interestingly, BRACO-19 did not perturb EBV-negative cell lines, suggesting specificity of this compound for an EBV target. Further analyses in a transient-transfection assay showed BRACO-19 reduced EBNA-1-dependent DNA replication via *ori*P and perturbed EBNA-1-mediated tethering to host chromosomes, a previously reported process essential for episome maintenance (33, 147). Overall, RNA G4s appear to be involved in maintaining EBV latency.

Like EBV, KSHV also maintains the viral episome in proliferating cells through the latency-associated nuclear antigen (LANA) protein (24). Here, LANA tethers newly replicated episomes to host chromosomes during mitosis (149). Specifically, LANA binds the GC-rich terminal repeats (TR) region of the KSHV genome, containing the *ori*P site for latent replication (150). The TR region is predicted to form several G4s, and oligonucleotides of these PG4s adopt a parallel topology (61). In KSHV-positive body cavity-based lymphoma (BCBL)-1 cells, the G4s co-localized with LANA in the nucleus, observed via immunofluorescence with the BG4 antibody. Treatment with PhenDC3, but not TMPyP4, reduced KSHV genome copies in cells (61), similar to the BRACO-19 induced phenotype in EBV (33). Yet the direct connection between this observation and LANA is still unclear.

#### Self-regulated EBV EBNA-1 and KSHV LANA expression

EBV EBNA-1 contains an essential glycine/alanine repeat (GAr) domain in between two glycine and arginine-rich domains and is used to evade host immune detection during latency maintenance (151). The GAr domain limits self-expression of EBNA-1 by suppressing its own corresponding mRNA sequence, of which 70% contains repeated PG4 sequences (152). CD analyses of GAr RNA PG4s exhibited parallel topology, confirming G4 formation *in vitro*. Furthermore, alteration or removal of these sequences increased EBNA-1 translation and antigen presentation in transfected cells. Incorporation of PDS into these experiments reproduced these findings. Overall, GAr RNA G4s assist in EBNA-1 self-regulation mechanisms.

Like EBNA1, the KSHV LANA mRNA sequence has several putative G4-forming sites (61). Treatment of either KSHV-positive primary effusion lymphoma BC3 B-cell line or BCBL-1 cells with TMPyP4 reduced LANA translation while LANA transcription and the expression of other latency-associated proteins remained normal (62). Like EBNA-1, LANA antigen presentation was also reduced upon G4 stabilization with TMPyP4. Therefore, LANA RNA G4s are likely involved in regulating LANA-mediated self-expression, similar to EBNA-1.

KSHV also contains microRNAs (miRNAs), non-coding RNAs known to regulate gene expression, termed miR-K12-1-9,11 (102). The miR-K12-1-9,11 gene cluster is located near latency-associated genes and is predicted to form G4s. Oligonucleotides of this gene sequence confirmed G4 formation *in vitro*, which adopted parallel topology. Using CD, PDS had minimal effect on G4 stability *in vitro*, while TMPyP4 produced a destabilizing effect. Mutation of the miR-K12-1-9,11 G4 sequence or treatment with TMPyP4 reduced promoter activity in transfected cells, indicating G4 formation may enhance miRNA promoter activity. The molecular mechanisms behind this are still unclear.

#### Non-self-regulated EBNA-1 and LANA expression

EBNA-1 expression and antigen presentation are also regulated by the host protein nucleolin (NCL) (64, 153). EBNA-1 interacted with NCL through the GAr domain (described above), both *in vitro* and in EBV-infected Mutu-1 cells (Burkitt lymphoma B cells) (64). The addition of the G4 stabilizer PhenDC3 precluded these interactions in both experimental systems, suggesting NCL interacts with the GAr through the EBNA-1 mRNA G4. In contrast, PDS did not perturb EBNA-1 GAr and NCL interactions or EBNA-1 expression levels. PDS also did not affect LANA expression or antigenic presentation in a later study with KSHV (154). The differences in chemical structure between PhenDC3 and PDS likely account for this, further verifying that not all commercial G4 stabilizers produce the same phenotype.

NCL also interacts with and modulates the expression of LANA, and like EBNA-1, NCL binds LANA mRNA G4s to suppress LANA translation (64). The G4 stabilizer PhenDH2 recapitulated these findings for both LANA and EBNA-1 (123). Another host protein, the heterogeneous nuclear ribonucleoprotein (hnRNP) A1, is an RNA-binding protein known to bind RNA G4s to regulate gene expression (155–157). hnRNP A1 also interacts with mRNA LANA G4s, and treatment with the G4 ligand TMPyP4 disrupts this interaction (62). It was proposed that hnRNP A1 may unfold LANA mRNA to increase LANA expression and the likelihood of immune detection by the host, yet this has not been definitively shown. Both hnRNP A1 and NCL also interact with HSV-1 G4s in an HSV-1-infected cell lysate pulldown assay, but the biological implications of this are unknown (54).

#### BHRF1 and KS-Bcl-2 dependent processes

The EBV and KSHV genomes encode a viral homolog of the human anti-apoptotic protein Bcl-2, named BHRF1 and KS-Bcl-2, respectively (158). Both are expressed early during lytic replication and regulate apoptosis, like the cellular Bcl-2, to promote survival of the infected cell (159). The gene sequence of *bcl-2* is reported to contain multiple PG4s at various promoter sites. Sequence analyses of the *bhrf1* and *KS-bcl-2* gene promoters showed only one *bhrf1* PG4 sequence, upstream of the transcription start site, while *KS-bcl-2* has multiple (69). The one EBV *bhrf1* PG4 is highly conserved across multiple EBV strains.

*In vitro*, the BRF1-G4 and a KS-bcl-2-G4 adopted a parallel topology, stabilized by PDS. In cells, both the BRF1 and KS-bcl-2 promoter activities were diminished when G4 formation was prevented via mutations, yet increased in the presence of PDS. This contrasts the human bcl-2 promoter, where G4 stabilization by PDS inhibits promoter activity, as observed by multiple groups (69, 160). Therefore, it could be that the G4-mediated expression of BRF1 or KS-Bcl-2 is used to promote viral replication by keeping the host cell intact.

### Lytic infection: *ori*Lyt dependent processes

An EBV PG4 identified downstream of *ori*Lyt, in an essential region for *ori*Lyt function (161), was found to adopt a parallel topology when examined as an oligonucleotide *in vitro* (56). Sequence analyses showed conservation with the corresponding region in HCMV, and promoter activity *in vitro* was not perturbed when the HCMV PG4 was replaced with the EBV G4. Furthermore, the HCMV replication factors, IE2, UL84, and UL44, all interacted with the EBV G4 in a pulldown assay, suggesting these conserved G4s share similar functions between subfamilies.

Most recently, a new EBV RNA transcript was identified that overlaps with the *IR4* region of the genome, shortly upstream of *ori*Lyt (122). It was found that this region of *IR4* can form G4s, allowing the complementary DNA strand to form a DNA:RNA hybrid that could be involved in regulating latent to lytic infection, yet more studies are needed to delineate how this process occurs at the molecular level.

Several G4s are predicted to form in the KSHV oriLyt sequence, and CD analyses confirmed this *in vitro* (71). It was found that RecQ1, a cellular helicase known to unwind G4s, bound an *ori*Lyt G4 site, and that either G4 stabilization with NMM or RecQ1 depletion reduced viral replication. Treatment of KSHV-infected BCBL-1 cells with either PhenDC3 or TMPyP4 reduced virion production and viral DNA replication. It was proposed that RecQ1 likely functions to unwind these oriLyt G4s to help facilitate lytic replication, as stabilization disrupts RecQ1 binding. As new lytic reactivation methods continue to develop, we anticipate G4s will show involvement in the initiation of lytic viral DNA replication, as seen in alpha- and betaherpesviruses.

## FUTURE OUTLOOK AND OUTSTANDING QUESTIONS

### How conserved are the roles of G4s in herpesviruses and beyond?

The effects G4 stabilizers have on herpesvirus lytic replication and latency, and what this means for viral G4 function, remain unclear. The current data presented herein suggest there are some overlapping functions among herpesvirus G4s, particularly in gene expression regulation, yet there is still much to be uncovered. The intricacies of working with herpesviruses in cell culture, which vastly differ between subfamilies, have resulted in most alphaherpesvirus G4 experiments focused on lytic infection, whereas gammaherpesviruses focus on latency. Potential targets include the LAT miRNA region in alphaherpesviruses and the HCMV IE1 tethering protein (162), and like gammaherpesvirus EBNA1 and LANA, are both enriched in PG4 sequences. Notably, G4 studies have not examined these processes in relevant latency models (neurons [163] or monocytes [164], respectively), and are needed to probe if G4s assist in regulating the balance between lytic and latent infection.

G4 sequence conservation among herpesviruses and the human host, with which herpesviruses have evolved for centuries (165), should also be explored, as this could improve G4 identification for antiviral purposes. Beyond herpesviruses, the roles of G4s in other viruses are also being investigated, and some share similarities with herpesviruses (166). Finally, G4s are not the only non-canonical nucleic acid structures. Other common examples include R-loops (167), RNA:DNA hybrids, and C-rich i-motifs, forming on the inverse side of a G4 or independently (168, 169). Both are predicted to form in herpesviruses (50, 170). Therefore, there is still much to be known about how non-canonical nucleic acid structures contribute to viral infections.

### Do G4 stabilizers reach the intended G4 target in cells?

Off-target effects and poor specificity remain key concerns in G4 antiviral discovery, as G4 ligands may bind both host and viral G4s. While competition mass spectrometry can assess binding affinity to specific G4 sequences (104), these experiments are performed *in vitro* and do not reflect cellular conditions. Ideally, G4 ligand binding should be mapped in infected cells. Techniques such as ChIP-Seq, CUT&Tag, and ChemMap enable G4 ligand binding determination, with the latter two applicable in live cells, using G4-specific antibodies or ligands (reviewed in reference 10). CUT&Tag was employed for HSV-1 (171) and *Mycobacterium tuberculosis* (172) with the BG4 antibody, yet other groups have reported non-specific binding artifacts with G4-specific antibodies when using CUT&Tag (173). Improving these methods will require different antibodies or labeled G4 ligands to enhance specificity. In contrast, probe-free methods such as G4access can assess G4 dynamics but do not map G4 ligand binding sites (100).

### How can G4 drug discovery be improved?

One major bottleneck in G4 research is that *in vitro* assessments of G4 formation often fail to reflect cellular phenotypes (174, 175), complicating *in vitro* high-throughput screening (HTS) approaches to identify effective G4 ligands. Cellular-based HTS screens could circumvent this (176) yet it requires sophisticated reagents, equipment, and method optimization. In contrast, bottom-up approaches are also limited due to minimal high-resolution structural studies of G4/target binding (175). Expanding these data sets, alongside molecular dynamics, structure-activity relationships (SARs), and machine learning predictions of biologically relevant PG4s, could improve target identification and ligand specificity (177). Continued advances in these areas will allow researchers to design G4 ligands with new chemistries, an effort already underway by other groups (66, 178–180), to improve current solubility and specificity issues.

Given the challenges listed above, there has yet to be an FDA-approved drug targeting G4s (174). Some G4 ligands have entered and failed clinical trials, including BRACO-19 (181), Quarfloxin (182), APTO-253 (183), and RHPS4 (184) for various issues surrounding toxicity, immune activation, and solubility. Despite this, the G4 stabilizer CX-5461 was given “fast track designation” by the FDA for patients with solid tumors (NCT02719977) (185) and QN-302 is in Phase 1 clinical trials against pancreatic cancer (NCT06086522) (186). The promising results with maribavir and CX-5461 against HCMV (78) suggest that combinatorial therapy should be considered as the field progresses.

### What is needed to move the herpesvirus G4 field forward?

**Fig 5:**
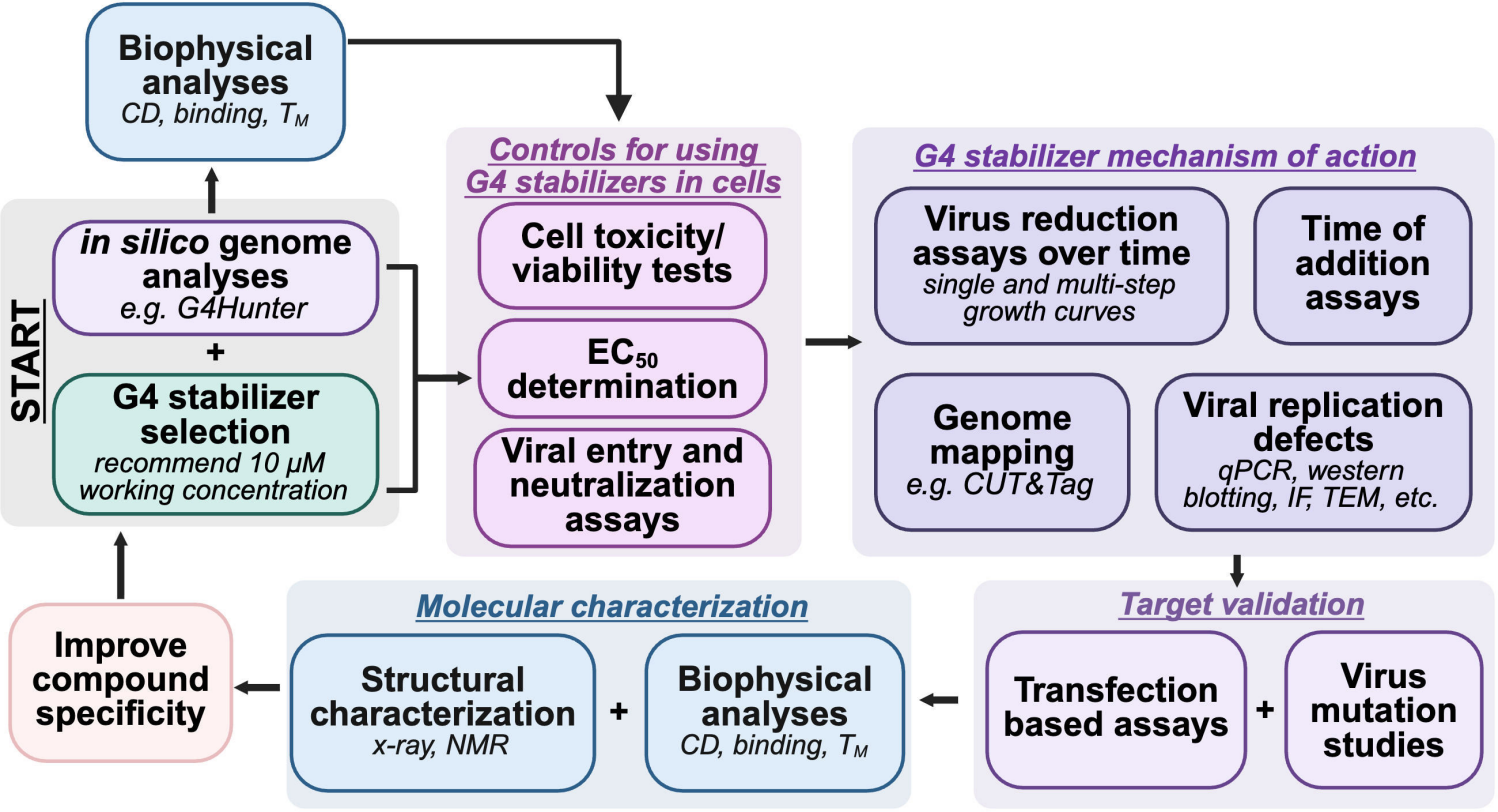
Recommended experimental flowchart for working with G4s in virally infected cells. When using a new G4 stabilizer, it is recommended to perform cellular controls and/or biophysical analyses with PG4 oligonucleotides before proceeding to cellular mechanistic studies and target validation. Additional biophysical analyses can be performed for molecular characterization and to improve compound specificity, if desired.

G4 stabilizers do not uniformly interact with G4s in specificity or binding mode, and this is largely due to the dynamic nature of G4s in cells. This is underscored by the fact that the same compound can produce different phenotypes depending on experimental conditions (Table 2). For example, BRACO-19 yields variable effects in HSV-1-infected cells depending on cell type and time post-infection (Table 2). Therefore, a systematic screening of G4 stabilizers during the different phases of lytic replication using standardized workflows is needed (Fig. 5). Although approaches may vary across subfamilies due to cell culture requirements, several key considerations should be kept in mind.

### G4 stabilizer choice, working concentrations, and stabilizer efficacy

Because G4 stabilizers vary in binding and specificity, each compound should be evaluated for cell toxicity and effective half-maximal (EC_50_) concentrations. Using compounds in cells well above the EC_50_ value risks saturation and non-specific binding, producing confounding results (187). Compound solubility should also be confirmed under working experimental conditions. Importantly, antiviral effects should not be assumed to result from viral G4 binding by the G4 stabilizer without proper validation. Viral entry and neutralization assays can exclude effects on early replication, while genome mapping approaches (e.g., CUT&Tag) can assess binding to viral and/or host sequences to aid in target validation.

### Assessment of G4 dynamics

Because cells, viruses, and G4s are dynamic, careful selection of cell lines and experimental time points is essential. Cancer-derived cell lines commonly used for G4 studies can be aneuploid or polyploid, adding another level of experimental heterogeneity. Additionally, U2-OS cells—frequently used in herpesvirus G4 studies—do not require the canonical viral transactivators (HSV-1 VP16/HCMV pp71) for gene expression (125, 126), potentially altering virus-mediated G4 regulatory processes. For example, in HSV-1, G4s putatively form near the VP16 consensus sequence and at the TATA box within IE promoters (53). However, the same G4s could be recruiting host proteins, such as SP1 (13, 74), complicating interpretations. Thus, drawing conclusions from both cancer-derived and primary cell models that support lytic replication is recommended. Although gammaherpesviruses remain challenging, emerging models that support lytic reactivation will help address these limitations (188, 189).

G4 dynamics during herpesvirus replication also remain underexplored. The BG4 antibody has confirmed G4 formation in HSV-1 and HCMV-infected cells, but integrating genome-wide approaches, such as CUT&Tag, across infection time points would better define viral and host G4 dynamics. Coupling these methods with G4 stabilizers could clarify compound specificity, localization, and effects on G4 formation while validating viral G4s as G4 stabilizer targets.

## CONCLUSIONS

Overall, the data presented herein demonstrate that herpesviruses employ G4s for various functions. In some cases, the effect of stabilizing viral G4s through exogenous ligands may be nullified due to the sheer number of G4s in the viral genome, making the antiviral properties less pronounced. This does not mean that viral G4s are not viable antiviral targets, but rather the current need for streamlined experimental workflows, improved G4/ligand site mapping, and new G4 ligand chemistries limits our understanding of G4s in herpesvirus biology. Furthermore, commercially available G4 stabilizers are powerful tools to assess G4 function in herpesvirus replication and beyond, yet care should be taken in addressing the nuances associated with the dynamic nature of G4s in cells. We anticipate that technological advancements in the field of G4 biology will continue to improve our understanding of these fascinating biological structures.
